# Information-Theoretic Neural Decoding Reproduces Several Laws of Human Behavior

**DOI:** 10.1162/opmi_a_00101

**Published:** 2023-09-20

**Authors:** S. Thomas Christie, Hayden R. Johnson, Paul R. Schrater

**Affiliations:** University of Minnesota, Minneapolis, MN, USA

**Keywords:** information theory, response times, rate coding, Hick-Hyman law, power law of practice

## Abstract

Human response times conform to several regularities including the Hick-Hyman law, the power law of practice, speed-accuracy trade-offs, and the Stroop effect. Each of these has been thoroughly modeled in isolation, but no account describes these phenomena as predictions of a unified framework. We provide such a framework and show that the phenomena arise as decoding times in a simple neural rate code with an entropy stopping threshold. Whereas traditional information-theoretic encoding systems exploit task statistics to optimize encoding strategies, we move this optimization to the decoder, treating it as a Bayesian ideal observer that can track transmission statistics as prior information during decoding. Our approach allays prominent concerns that applying information-theoretic perspectives to modeling brain and behavior requires complex encoding schemes that are incommensurate with neural encoding.

## INTRODUCTION

Human response time during task performance is one of the most widely studied phenomena in behavioral psychology. The time it takes to respond to a stimulus provides a measurable behavioral window into otherwise hidden cognitive processes, facilitating insight into diverse aspects of cognition such as priming effects (Hart et al., 2010), the rate of learning from practice (Newell & Rosenbloom, 1981), the impacts of switching tasks (Draheim et al., 2016), and the interaction of conflicting or ambiguous stimuli (MacLeod, 1991).

The response time literature reveals reproducible patterns across subjects and tasks. Distributions of response times tend to be positively skewed, resembling log-normal or gamma distributions (Lindeløv, 2019; Ratcliff, 2012). Response times increase reliably with task complexity as described by the Hick-Hyman law (Hick, 1952; Hyman, 1953). Responses also quicken as a function of practice, a phenomenon called the power law of practice (Newell & Rosenbloom, 1981). The Stroop effect famously captures the slowing of response times (with a concomitant decrease in accuracy) when a subject is asked to override a habitual or “pre-potent” response (MacLeod, 1991; Stroop, 1935). Each of these phenomena has been thoroughly replicated and modeled in detail, with several distinct explanations for why each might arise. However, a unified parsimonious explanation of these phenomena, describing them jointly as necessary outcomes of some process or constraint on cognition, has not yet been proposed.

In this article, we fill this explanatory gap by showing that each of these phenomena arise in a simple model of neural decoding, namely, as the time it takes to infer messages encoded in an array of Poisson processes. A Poisson process generates a series of events whose times are uncorrelated and are unpredictable in the short term, though they follow a predictable average rate *λ*. Poisson processes are often used as a simple model of neural firing, where each event is treated as a spike time for a neuron (Heeger, 2000). In a neural rate code, properties of stimuli or tasks are encoded as patterns of firing rates over population of neurons. When information is encoded this way, we can ask: how long does it take to determine which pattern of rates are generating the observed spikes, and therefore what is being encoded? When decoding is performed by an ideal observer, we will show that decoding duration produces patterns that qualitatively match the response time phenomena mentioned above. As human response times require information transmission using neural rate codes, decoding time serves as a bound for response time.

In the context of other models of information transmission and decoding, the paradigm we propose here has three distinguishing features. First, information transmission occurs in continuous-time. Most applications of information theory encode messages as sequences of symbols with discrete indexes, or as a sequence of “blocks” (Cover & Thomas, 2012). As we will show, the proposed model has the decoder observe the array of Poisson processes in real time, continuously updating its belief about probable messages (see Figure 2D).

Second, the decoder tracks its decoding confidence by computing the self-entropy over a distribution of possible messages, as shown in Figure 2E. A decrease in entropy is equivalent to information gain by the decoder. Entropy naturally captures the decoder’s uncertainty over messages and can be easily computed, even when the set of possible messages is large. When the decoder’s entropy reaches a pre-set threshold, the most likely message is taken to be the true encoded message.

Third, the decoder maintains a prior belief over possible messages, which is combined with the observation likelihoods to produce a posterior belief over which the entropy is calculated. This allows the decoder to exploit task statistics during decoding — more likely messages tend to be decoded more quickly. In most information-theoretic encoding schemes, for example Huffman coding or adaptive coding (Vucetic, 1991), message statistics are used to construct complex encoding schemes to achieve maximum transmission efficiency. The use of complex encodings has sparked debate on the applicability of information theory to the study of the brain and behavior (Laming, 2010; Luce, 2003). Our model, on the other hand, places this optimization process in the decoder, implemented as a simple vector of prior beliefs over messages rather than a complex algorithm. In this way, message transmission can leverage prior beliefs to speed transmission without relying on long “blocks” of symbols or complex encoding schemes.

In what follows, we describe the proposed model in detail, showing through simulations how the model produces the patterns of response times mentioned above, and how the patterns depend on the various model parameters and constraints. We then explore the possibility of learning an efficient codebook using reinforcement learning, finding that transmission time is minimized when the activation of the simulated neurons is sparse. Lastly, we discuss how our model relates to other approaches to response time modeling and the general debate around the utility of information theory to the study of the brain and behavior.

## THE MODEL

**Figure 1.**
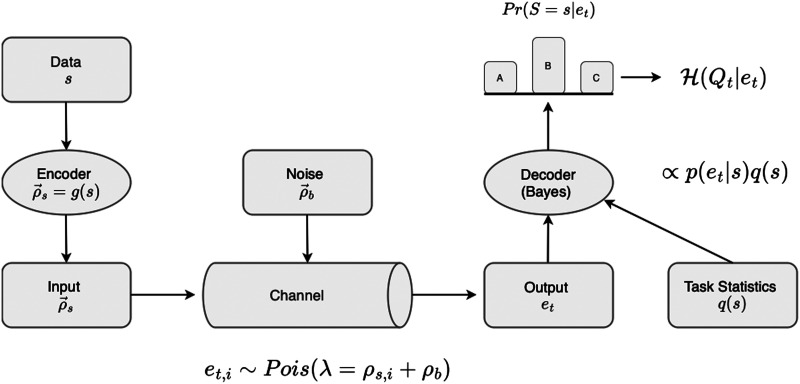
Information transmission is structurally similar to the channel model introduced by Shannon (Shannon, 1948). The encoder maps symbols *s* ∈ *S* to vectors of firing rates $\vec{\rho_{s}}$, with individual Poisson processes indexed by *i*. A shared noise rate *ρ*_*b*_ is added, then individual events are generated by each Poisson process *i* with rate *λ* = *ρ*_*s*,*i*_ + *ρ*_*b*_. The decoder observes the events at time *t* and calculates a posterior probability *Pr*(*S* = *s*|*e*_*t*_) that the events were generated by each possible symbol *s* ∈ *S*. The decoder’s posterior belief incorporates both the observations made and the prior belief about the relative frequency of transmission each symbol. Finally, the decoder computes the entropy of the posterior over symbols 𝓗(*Q*_*t*_|*e*_*t*_) and compares it to a stopping threshold.

In the model we propose, an encoder encodes discrete symbols into an array of Poisson process rates with noise added. A decoder observes events produced by the Poisson processes and decides which symbol is being encoded. This scenario is illustrated in Figure 1. To be more explicit, suppose the environment presented stimuli *s* ∈ *S* to an encoder with some probability *Pr*(*S* = *s*), which we will denote *p*(*s*). Let *N* be an array of *n* Poisson processes, or simulated neurons, with a baseline firing rate *ρ*_*b*_^1^. We can write the baseline firing rates as an *n*-length vector $\vec{\rho_{b}}$ = [*ρ*_*b*_, *ρ*_*b*_, …]. This vector represents the channel’s noise. An encoder *g* encodes a message *s* by increasing the firing rate of each neuron in *N* by some value, written as an vector $\vec{\rho_{s}}$ = [*ρ*_*s*,1_, *ρ*_*s*,2_, …, *ρ*_*s*,*n*_]. The total firing rate of the vector of neurons encoding message *s* is then $\vec{\rho_{b}}$ + $\vec{\rho_{s}}$ = [*ρ*_*b*_ + *ρ*_*s*,1_, *ρ*_*b*_ + *ρ*_*s*,2_, …, *ρ*_*b*_ + *ρ*_*s*,*n*_]. This vector represents the channel’s signal.

**Figure 2.**
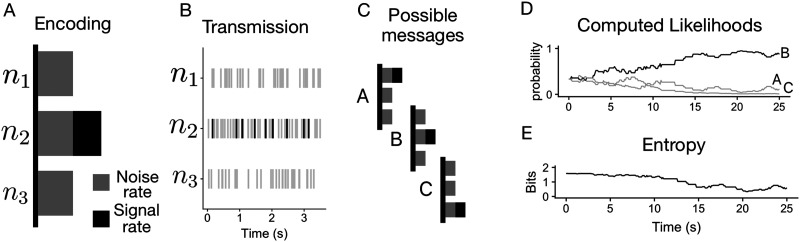
Schematic of a message transmission. (A) A population of 3 neurons each has a baseline noise rate. A codebook maps each message to a vector of signal rates, which are added to the noise rates. (B) Each neuron fires according to a sum of its signal (black) and noise (gray) rates. Dashes represent spikes. (C) A codebook, available to both the encoder and decoder, maintains the firing rates associated with each possible message. The decoder knows the baseline noise rate. (D) The decoder computes the likelihood that the observed spikes were generated by each message. (E) The entropy of the posterior distribution over messages is computed at each timestep. When the entropy reaches a pre-specified threshold, the most likely message is taken to be the true transmitted message. A lower entropy threshold will increase decoding accuracy at the expense of decoding time.

For example, suppose the population of neurons was size *n* = 3 and a signal *s*_1_ had a corresponding signal vector $\vec{\rho_{s_{1}}}$ = [0, 4, 0]. Suppose further that the baseline noise rate was *ρ*_*b*_ = 6. Then the overall firing rate, in spikes per second, would be $\vec{\rho_{s_{1}}}$ + $\vec{\rho_{b}}$ = [6, 6 + 4, 6] [(6 + 0), (6 + 4), (6 + 0)] = [6, 10, 6]. This scenario is represented in Figure 2A.

Let the set of symbols and corresponding “signal vectors” be stored in a codebook *C* (Figure 2C). The job of the decoder is to continually watch the set of spikes emitted by the neurons and determine which message, of all possible messages, is being encoded. We will assume that the decoder has access to the codebook *C* of possible messages and their corresponding signal vectors. It also knows the baseline noise rate *ρ*_*b*_. Furthermore, we will assume that the decoder has a perfect memory and can keep an accurate running tally of spikes emitted by each neuron between time *t*_0_ and time *t*, call these *e*_*t*_ = [*e*_1,*t*_, *e*_2,*t*_, …, *e*_*n*,*t*_] At any point in time, the decoder can use this information to compute the likelihood *Pr*(*S* = *s*|*e*_*t*_) = *p*(*s*|*e*_*t*_) of the number of observed spikes given each message, as shown in Figure 2D. Due to the decoder’s perfect memory, access to the codebook and noise rate, and ability compute a Poisson likelihood, we consider the decoder an ideal observer^2^.

How long will this ideal decoder take to infer which signal *s* is being encoded? The decoding cannot be instantaneous: in zero time, zero spikes will have been observed. The random nature of the observations, generated as they are by Poisson processes, means that the decoder should never be 100% confident in any decoding judgment, no matter how much time has passed. Taken together, these imply that the decoder should observe spikes emitted from the encoder until it is sufficiently confident in the encoded symbol, or said another way, until its uncertainty about the symbol value is sufficiently reduced. As is common in models of information transmission, we use the self-entropy 𝓗 to compute uncertainty.

The decoder’s uncertainty depends on the likelihood of the observations, but it also depends on the decoder’s prior belief about how likely each symbol is to be transmitted, call this *Pr*(*S* = *s*) = *q*(*s*). Note that the true transmission frequency *p*(*s*) and the decoder’s belief about transmission frequency *q*(*s*) may be different distributions. The decoder uses Bayes’ rule to compute a posterior belief over possible messages, *Pr*(*S* = *s*|*e*_*t*_) ∝ *p*(*e*_*t*_|*s*)*q*(*s*) = *q*(*s*|*e*_*t*_), using *q* to indicate that the value includes the decoder’s (possibly incorrect) prior beliefs. Let the vector of posterior probabilities at time *t* be referred to as *Q*_*t*_, with an initial entropy

$$𝓗\left(Q_{0}\right)=\sum_{s\in S}q\left(s\right)\log\;q\left(s\right)$$

The decoder’s uncertainty about which symbol generated the observed spikes is computed as

$$𝓗\left(Q_{t}|e_{t}\right)=\sum_{s\in S}q\left(s|e_{t}\right)\log\;q\left(s|e_{t}\right)$$

The time-course of the conditional entropy 𝓗(*Q*_*t*_|*e*_*t*_) in an example transmission is shown in Figure 2E. The entropy over posterior message probabilities is updated until it reaches a pre-specified threshold. When the threshold is reached, the inferred message is the message with the greatest posterior probability. The decoding accuracy is determined by whether the decoder’s judgment about the most likely encoded signal is correct. When there are multiple choices, this can be understood as a model of forced-choice reaction times.

## AN INSTANTIATED EXAMPLE WITH SIMULATIONS

Our goal in this section is to demonstrate via simulations the relationships between patterns of decoding times the model described above and patterns of response times described in the literature. In particular, we show that a very simple model can parsimoniously account for a range of phenomena. We instantiated the model in both R and Python. Code used to reproduce the results in this section is available at https://github.com/tom-christie/transmit.

Our interest in the current paper is in investigating qualitative similarity rather than quantitative model fitting. To that end, we keep the model and encoding scheme as simple as possible. With the exception of our analysis of sparse coding, the codebook consists of 1-hot sparse codes, with a pre-specified noise rate *ρ*_*b*_ > 0 and where the signal rate for a single neuron *ρ*_*s*,*i*_ > 0 and *ρ*_*s*,*j*_ = 0 for all other neurons *j* ≠ *i*. Despite our use of Poisson processes, the model presented here is not a model of neural activity; rather, it is a toy model designed to show that a wide range of phenomena can, in principle, arise from basic principles.

### Decoding Time Distribution

**Figure 3.**
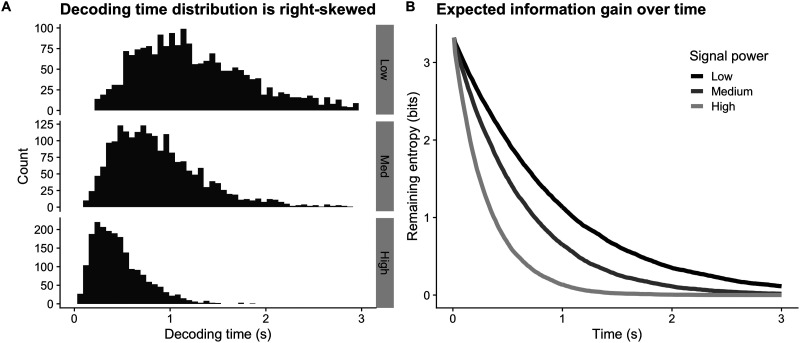
2000 simulated transmissions were performed with a baseline noise rate *ρ*_*b*_ = 10, three signal powers (Low = 8, Medium = 10, and High = 15), an entropy threshold of 0.5 bits, and 10 possible messages. (A) Decoding time distributions are right-skewed, similar to human behavior. Each panel shows a different value for signal power, with higher signal power generating faster decoding time, on average. (B) Average entropy across simulations is plotted at each point in time, for each value of signal power. The decoder begins with an initial amount of uncertainty (*log*_2_(10) bits) which decreases over time as observations are made. A larger signal-to-noise ratio results in faster inference. Information gain is not linear with time, but instead shows smaller marginal gains as time progresses.

Human response times are characteristically noisy, varying substantially even with repeated presentations of the same stimuli. This variability is well-modeled by both Wald and shifted log-normal distributions (Lindeløv, 2019; Ratcliff, 2012). Figure 3A shows a characteristic distribution of decoding times produced by the model. Our “channel” consisted of a population of 10 neurons with a baseline noise rate of *ρ*_*b*_ = 10, a codebook with 10 possible messages (encoded as a 1-hot vector where each neuron corresponds to a symbol), with three different signal powers, Low (8 spikes/second), Medium (10 spikes/second), and High (15 spikes/second). The entropy threshold was set at 0.5 bits, and the decoder utilized a uniform prior over possible symbols, with a corresponding initial entropy of log_2_ 10 bits. The plot shows the distribution of decoding times for 2000 transmissions for each level of signal power.

As the decoder observes spikes, its belief about the symbol being encoded changes over time, as shown in Figures 2D and E. Each individual time course is noisy, similar to the Wiener process used in a drift diffusion model (Ratcliff et al., 2016). However, averaging over many transmissions gives a clear picture of the expected rate of information gain, plotted in Figure 3B. The plot reveals an important component of this model: information gain is not constant over time. The decoder’s uncertainty is reduced with continued observation, but the investment has diminishing returns.

### The Hick-Hyman Law

One of the earliest and most controversial applications of information-theoretic concepts to human behavior was the discovery by William Hick and Ray Hyman that response times vary with the amount of information provided by a stimulus. In an experiment requiring a subject to map stimuli to behavioral responses, information about the correct response is encoded as the number of distinct stimuli (Hick, 1952), for example, or the stimuli's relative probabilities (Hyman, 1953). The legitimacy of an information-theoretic analysis of this finding has been regularly disputed in the years since (Laming, 2010; Luce, 2003). Criticizing Hick’s interpretation of his findings, Laming said the idea that “the relationship between stimuli and reaction time is that dictated by the capacity limitation of a communication channel … will not wash. A choice-reaction experiment involves the transmission of single stimuli, one at a time, a condition that affords no opportunity for the sophisticated coding on which Shannon’s theorem depends” (Laming, 2010, pp. 734–735).

Though neither author states it directly, Laming and Hick are likely referring to Shannon’s Source Coding Theorem, which states that data compression (and therefore transmission time) is bounded from below by the entropy of the source producing symbols to be transmitted. The entropy of the source, of course, is linearly related to the logarithm of the number of equally likely possible symbols. To our reading, Laming is criticizing Hick’s inference that the logarithmic relationship between response time and the number of options is an outcome of this relationship because the relationship can only hold if the encoding were transmitted while maximally compressed. Compression, in turn, requires knowledge of symbol statistics and a complex encoding scheme. Or does it?

**Figure 4.**
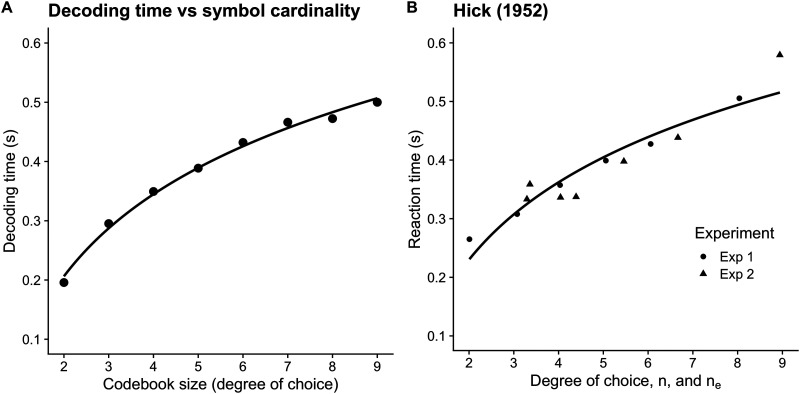
Transmission time as a function of codebook size and information transmitted. (A) We simulated 2,000 symbol transmissions with codebooks of varying sizes with noise rate *ρ*_*b*_ = 10, signal power *ρ* = 18, and an entropy threshold of 0.5 bits. Black dots show the average decoding time over transmissions, and the black line represents a logarithmic model fit. As in Hick’s experiments, transmission time increases with the logarithm of the number of possible messages. (B) Response times re-plotted from Hick (1952).

To investigate the relationship between decoding time and the number of possible symbols, we transmitted symbols while varying the cardinality of *S*, the set of symbols. As before, the decoder used a uniform prior over possible symbols. The results shown in Figure 4A reveal a logarithmic relationship between the number of possible symbols and decoding time. This matches Hick’s finding, re-plotted from Hick (1952) in Figure 4B and fitted with a logarithmic curve. Importantly, our model produces this relationship *without* sophisticated encoding schemes or the transmission of many messages together in a long block. It is worth considering how this is possible.

Hick interpreted his data as suggesting that information is transmitted from stimulus to response at a constant rate, which manifests as a logarithmic relationship between the number of possible stimuli and response time. Our model reveals the same relationship. At first glance, this result seems to contradict our claim above that information transmission is not linear with time, but has diminishing returns. This confusion is an opportunity to make a subtle but important point. In our model, indeed, information transmission (as captured by uncertainty reduction) is not constant over time while decoding a single symbol. However, decoding time *does* appear to be linear with the entropy of the decoder’s prior belief about message symbols *across tasks*, or said another way, across codebooks. For each task, this relationship is dependent on the decoder’s prior belief over symbols, because the prior (rather than the source statistics) defines the effective entropy of the symbols. As it is a function of the decoder’s belief, entropy is a *subjective* value. If the decoder’s belief about the relative likelihood of symbols changes, we should expect decoding times to change accordingly. Indeed, our results in Figures 6A and 7A, discussed in further detail below, reflect exactly this. In this way, information about task statistics is exploited in a relatively simple way by the decoding process, rather than through a complex encoding scheme.

We cannot know the prior belief of the subjects in Hick’s experiment, but we do know they practiced the task extensively. Hick asked subjects to practice each condition many times; the reported number is at least 8,000 on his first experiment. He also reported practice effects in Figure 3 of his (Hick, 1952) paper, and it is easy to see from that figure that the logarithmic relationship only appears after practice. Practice allows the subject to learn a task-specific prior belief about the stimulus. It appears that as it relates to response time, this belief *must* be learned through practice rather than explicit instruction. Despite being aware that there are e.g., 10 bulbs to choose from, the subject behaves *as if* there are many more possibilities, as reflected by their response time, until practice is achieved and the probability mass on the decoder prior rests heavily on 10, and only 10, options. Viewed a certain way, practice can be seen as a mechanism for compressing stimulus features until only those most relevant to the task at hand are considered as plausible “symbols” to be decoded. Once this has taken place, the number of possible stimuli does appear to vary logarithmically with response time.

Several questions arise naturally at this point: What if stimuli have unequal probability? What if the decoder’s prior belief is incorrect? And finally, at what *rate* do response times achieve this apparently efficient value as a function of practice? We will address each of these in turn.

### Transmission Time Is Linear With Surprisal

While Hick’s findings describe a relationship between the number of stimuli and response time, Ray Hyman varied the statistics of stimuli within the task, both by modifying the relative frequency of stimulus options and the conditional probabilities of sequences of stimuli (Hyman, 1953). Hyman’s results replicate and extend Hick’s main finding. The paper highlights that response time is a function not only of the number of possible stimuli, but also of the subject’s *belief* about the relative probabilities of the stimuli, specifically the stimulus surprisal.

**Figure 5.**
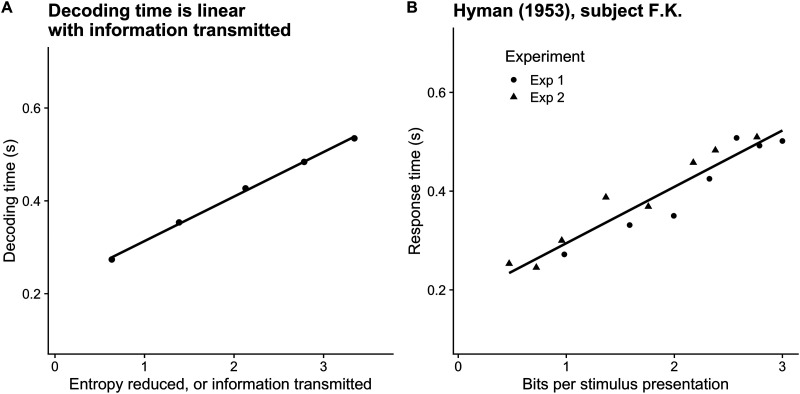
Transmission time for individual messages with unequal frequency varies linearly with the amount of information transmitted. (A) We simulated 50,000 transmissions of five symbols with approximate probabilities [0.5^1^, 0.5^2^, 0.5^5^, 0.5^4^, 0.5^5^], re-normalized to sum to 1, with an entropy threshold of 0.7 bits, noise threshold *ρ*_*b*_ = 10, and signal power *ρ* = 12. This plot shows the information gain from each transmission, calculated as *D*_*KL*_(*p*(*s*|*e*_*t*_)‖*p*(*s*)), or the KL-divergence between the prior distribution over messages and the posterior at decision time. Here, the decoder’s prior distribution matches the true message distribution. For the frequent symbol A, the prior is ‘closer’ to the posterior at decoding time as measured by KL-divergence so the message carries less surprisal, while the message D is believed to be less frequent and is thus more surprising to observe. (B) Response times from subject F. K. are re-plotted from Hyman (1953). Experiment 1 replicated Hick’s manipulation of the number of possible stimuli, while in Experiment 2 Hyman varied the relative frequency of stimuli.

We simulated Hyman’s manipulation by transmitting four symbols with a non-uniform distribution and recording decoding times. In each case, the decoder had accurate prior knowledge of the relative message frequencies. As suggested above, this is our model’s equivalent to a subject having had extensive practice at a task—and indeed, Hyman reports over 15,000 reaction times recorded over a course of 3 months for each subject. Figure 5A shows response time as a function of surprisal for a decoder. Each point indicates the average decoding time of one symbol. The vertical axis records the decoder’s information gained via observations *e*_*t*_ at the time the decoding decision is made, averaged over many transmissions:

$$\text{Information}\;\text{gained}=\text{E}\left[D_{KL}\left(q\left(s|e_{t}\right)\|q\left(s\right)\right)\right]$$

The prior over symbols, *q*(*s*), is the same for each transmission, but the posterior has its probability mass re-weighted over symbols, with the most mass on the most likely symbol given the observation. When a rare symbol — or a symbol *believed* to be rare — is being decoded, the divergence between the prior belief and the belief at decoding time is large. It takes longer for this symbol to be decoded than a likely symbol, as it takes longer to collect sufficient evidence to overwhelm a skeptical prior. This is reflected in the decoding time, which once again appears to be linear with information gain. To reiterate, this relationship holds across messages, but not within individual transmissions.

**Figure 6.**
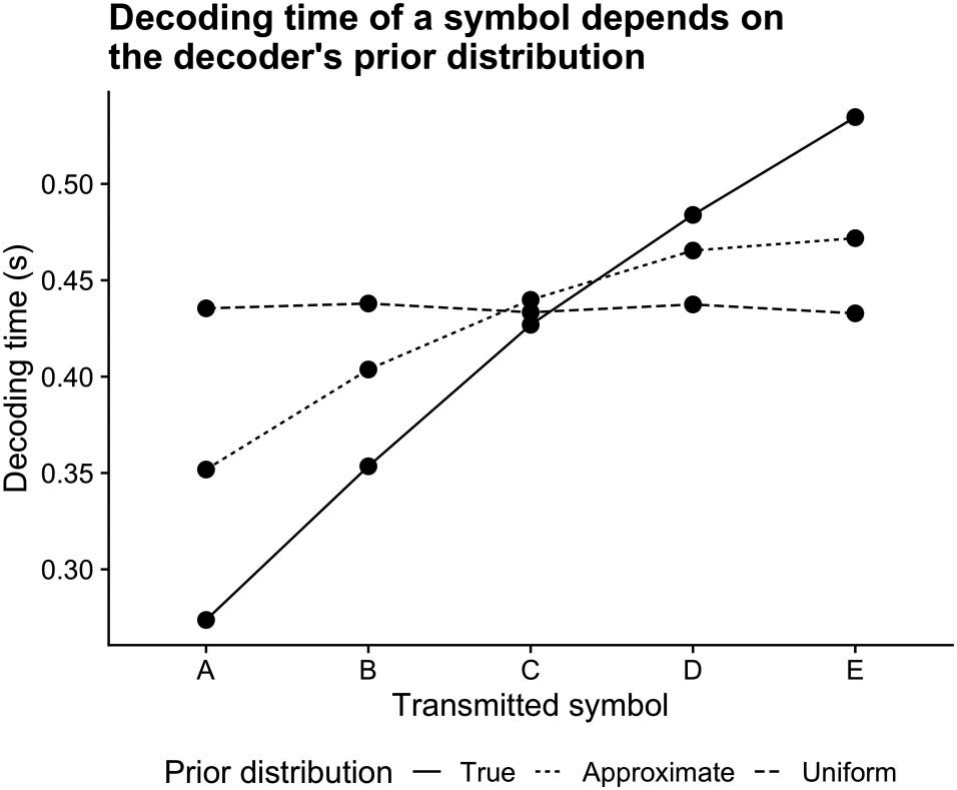
We transmitted five symbols with decreasing order of frequency, where A is more frequent than B, etc. Despite the non-uniform symbol distribution, a uniform prior over symbols (dashed line) leads to equivalent decoding times for each symbol. As the decoder’s prior belief is updated and more closely resembles the true source distribution, more frequent symbols are decoded more rapidly, and less frequent symbols less rapidly, leading to an overall decrease in the expected decoding time. 50,000 transmissions were performed for each symbol and prior distribution condition. As in Figure 5, symbols had transmission frequencies [0.5^1^, 0.5^2^, 0.5^5^, 0.5^4^, 0.5^5^], re-normalized to sum to 1, with entropy threshold of 0.7 bits, noise threshold *ρ*_*b*_ = 10, and signal power *ρ* = 12. The “Approximate” prior had values [0.360, 0.228, 0.168, 0.065, 0.032].

Decoding time is a function of the decoder’s prior belief about message frequency rather than the probability of messages defined by the symbol generating process. Accordingly, a uniform prior over messages with very different source frequencies will result in uniform decoding times. As the decoder’s prior changes, so does the decoding time for messages: messages believed to be more likely will be decoded more quickly, because less evidence is needed to “convince” the decoder of the signal’s content. This relationship is illustrated in Figure 6. We ran repeated simulations, simulating five symbols with the same uneven relative frequency while varying the decoder’s prior beliefs.

### The Power Law of Practice

If the decoder’s prior belief about message frequency influences decoding time, we should be able to minimize decoding time by optimizing the prior to match the true frequency of symbol transmissions. Efficient coding systems, such as Huffman coding, achieve optimal codes by perfectly matching an encoding scheme to the statistics of the messages to be encoded. In practice, however, perfect knowledge of the message statistics is often unavailable and must be estimated and continually updated from observations.

Suppose a person began practicing an unfamiliar task with many trials. In the beginning, they would have very uninformative, mild beliefs about task statistics: they could not know which stimuli occur at what rate, or even which features of the environment are task-relevant. During the course of practice, two things happen: the person would narrow their focus to only task-relevant stimuli (Dayan et al., 2000), and they would observe the empirical frequencies of those stimuli. An ideal observer with perfect memory could keep a running record of each stimulus and compute its relative frequency, continually updating a prior belief about the likelihood of subsequent stimuli. As many stimuli are observed, this belief would slowly approach the actual source frequency of the stimuli. Using the model we propose here, the updated prior belief corresponds to improved response times.

Acquiring task statistics in this way is bounded by subject participation in trials. In our model, this is analogous to the decoder receiving messages in sequence. Decoding times are improved by an improved estimate of symbol statistics, but this can only happen at a rate bounded by the process by which observations of trial stimuli are incorporated into the decoding prior.

We ran simulations to investigate the rate of response time improvements as a function of sequential message transmissions. We started 1,000 naive decoders with uniform prior distributions over symbols. We simulated message transmission time across decoders after many trials, ranging from 1,500 to 75,000 trials. Each decoder maintained task statistics as the parameters of a Dirichlet distribution with the same length as the number of possible symbols. After each transmission, the decoder incremented the corresponding parameter by a value of 1, then the mode of the distribution was used as the prior for the subsequent message.

**Figure 7.**
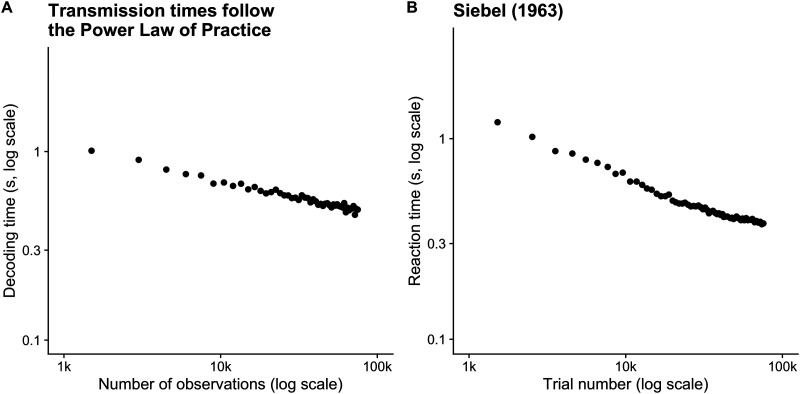
Symbol transmission time decreases with practice, or, as the decoder develops an improved estimate of message statistics. Simulated transmission times closely match human results. In both cases, the logarithm of the number of practice trials is linearly related to the logarithm of transmission/response time, a phenomenon known as the power law of practice. (A) We repeatedly transmitted two symbols from a possible codebook of cardinality 10. The decoder began the task with a Dirichlet prior with each parameter equal to 1,000, capturing a strong belief in a uniform symbol frequency. Each point represents the mean decoding time of 1,000 transmissions with the same prior distribution. We then incremented the decoder’s prior belief to capture the observed message statistics (sampled from a multinomial distribution) and re-transmitted another 1,000 messages. For example, after 1,500 observations, the expected value of the Dirichlet parameters was updated from [1*k*, 1*k*, 1*k*, 1*k*, …] to [1.75*k*, 1.75*k*, 1*k*, 1*k*, …]. Each point in the figure represents 1,500 more trials. As more and more trials are performed, the mode of the Dirichlet distribution, which is employed by the decoder as a prior belief, more closely matches the true transmission statistics. Each transmission had an entropy threshold of 0.5 bits, a noise rate of *ρ*_*b*_ = 10, and a signal power *ρ* = 8. (B) Human response times re-plotted from Newell and Rosenbloom (1981), which in turn is re-plotted from Seibel (1963). Each point represents the average response time over many subjects after the specified number of trials.

Simulation results are shown in Figure 7A, plotted next to experimental data on a digit span task from Seibel (1963). The simulated messages are decoded faster as the number of trials increases, despite using the same encoding process in each trial. The linear relationship in the log-log plot between practice trials and decoding time is characteristic of the power law of practice, a phenomenon widely observed in behavioral tasks (Newell & Rosenbloom, 1981). The experimental data shown in Figure 7B shows results from a single experiment, but different experiments result in the same characteristic line but with varying slopes. In our model, an update to the prior of less than one alters the slope of the line and the rate of learning and may be optimal when updates are costly (Lieder et al., 2018).

The power law of practice (also called the power law of learning) has been criticized as describing aggregate behavior over subjects rather than the expected learning rate of any individual (Heathcote et al., 2000). We agree, and note that the results in Figure 7A are aggregated across 1,000 decoders, with each decoder updating its own prior *q*(*s*). We would expect each decoder to start with a distinct value for *q*(*s*) and maintain a distinct learning profile that is characteristic of the specific sequences of messages the decoder has received.

### Speed-Accuracy Trade-Off

**Figure 8.**
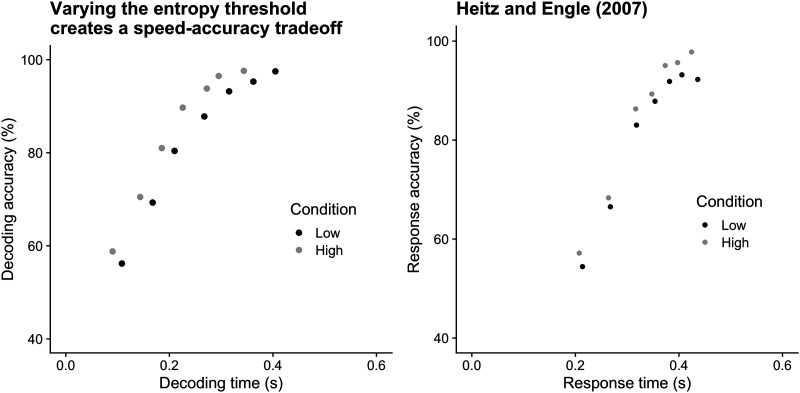
(A) Decoding time is a function of the decoder’s entropy threshold. Varying the entropy threshold produces distinct speed-accuracy-tradeoff curves. Each dot is the average decoding time of 1,000 transmissions with entropy thresholds ranging from 0.2 to 2.5 bits and a codebook of size 10. A noise power of *ρ*_*b*_ = 8 was used. Black dots used a signal power of *ρ*_*low*_ = 18, and gray dots used *ρ*_*high*_ = 20. (B) Human response times re-plotted from Heitz and Engle (2007).

Varying the entropy stopping threshold produces a speed-accuracy trade-off curve qualitatively similar to those observed in behavioral experiments in humans (Heitz, 2014) and monkeys (Hanks et al., 2014), resulting in decodings that are either fast and inaccurate, or slow and accurate. Separate curves are often observed across subjects or between conditions in experiments, which we simulated by using two values for signal power, the maximum rate at which neurons carrying the signal can fire. Decoding times are shown in Figure 8A, compared with experimental data from a working memory task.

### Congruence and Conflict: The Stroop Effect

So far we have analyzed tasks in which stimuli, or symbols, are transmitted one-at-a-time. However, tasks can be complex and multiple stimulus attributes can carry information relevant to action selection. When attributes lead to the same action, they are often called “congruent”, while attributes predicting different actions are called “conflicting”. In the famous Stroop task, for example, subjects are asked to state the color of ink in which a word is written. The color and text of printed words can be congruent (the word ‘green’ written in green ink), incongruent (‘green’ written in red ink), or neutral (‘green’ written in black ink). When compared to the neutral condition, response times are characteristically faster when stimulus properties are congruent, and slower when incongruent (Stroop, 1935).

**Figure 9.**
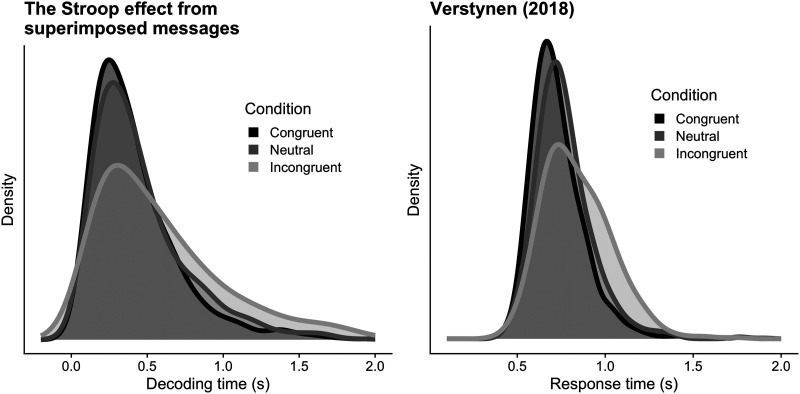
(A) Superimposing multiple signals from the codebook produces variations in decoding time that mirror the Stroop effect. We transmitted 5,000 transmissions for each of 3 conditions, with noise power *ρ*_*b*_ = 12, signal power *ρ* = 12, and an entropy threshold of 0.1 for a codebook with only two symbols. In contrast to the transmissions in other tasks, here we superimposed the rate vectors for more than one symbol for each transmission. Specifically, the neutral condition for symbol 1 had a rate vector $\vec{\rho }$ = [12 + 12, 12], the congruent condition had $\vec{\rho }$ = [12 + 12 + 3, 12], and the incongruent condition had $\vec{\rho }$ = [12 + 12, 12 + 5]. The additional (congruent) 3 and (incongruent) 5 are fractional additions of the vectors associated with the same and different symbols, respectively. (B) Human response time distributions re-plotted from Verstynen (2018).

We modeled the simultaneous transmission of two symbols (called multiplexing) creating an encoding consisting of a linear combination of the constituent rate vectors. Resulting response distributions are shown in Figure 9A, and strongly resemble experimental data in Figure 9B.

## LEARNING AN EFFICIENT CODE

While the above results prioritized simplicity by mapping discrete signals to 1-hot vectors of firing rates, we additionally investigated the characteristics of learned codebooks. To do this, we used a reinforcement learning algorithm to learn a codebook that minimized a combination of decoding accuracy, decoding time, and the number of spikes during transmission. In this paradigm, the agent acts as both transmitter and decoder and is tasked with learning a policy (encoding) that maps states (symbols) to actions (*n*-length vectors of firing rates). This mapping was available to the decoder during decoding, consistent with our model assumptions above.

We implemented the algorithm using the TD3 algorithm (Fujimoto et al., 2018) as implemented in the stable-baselines3 package (Raffin et al., 2021). The TD3 algorithm is an extension of the DDPG algorithm (Silver et al., 2014), in which the agent concurrently learns a Q-function (value of state-action pairs) and a policy (mapping of observations to actions) in order to maximize the reward.

**Figure 10.**
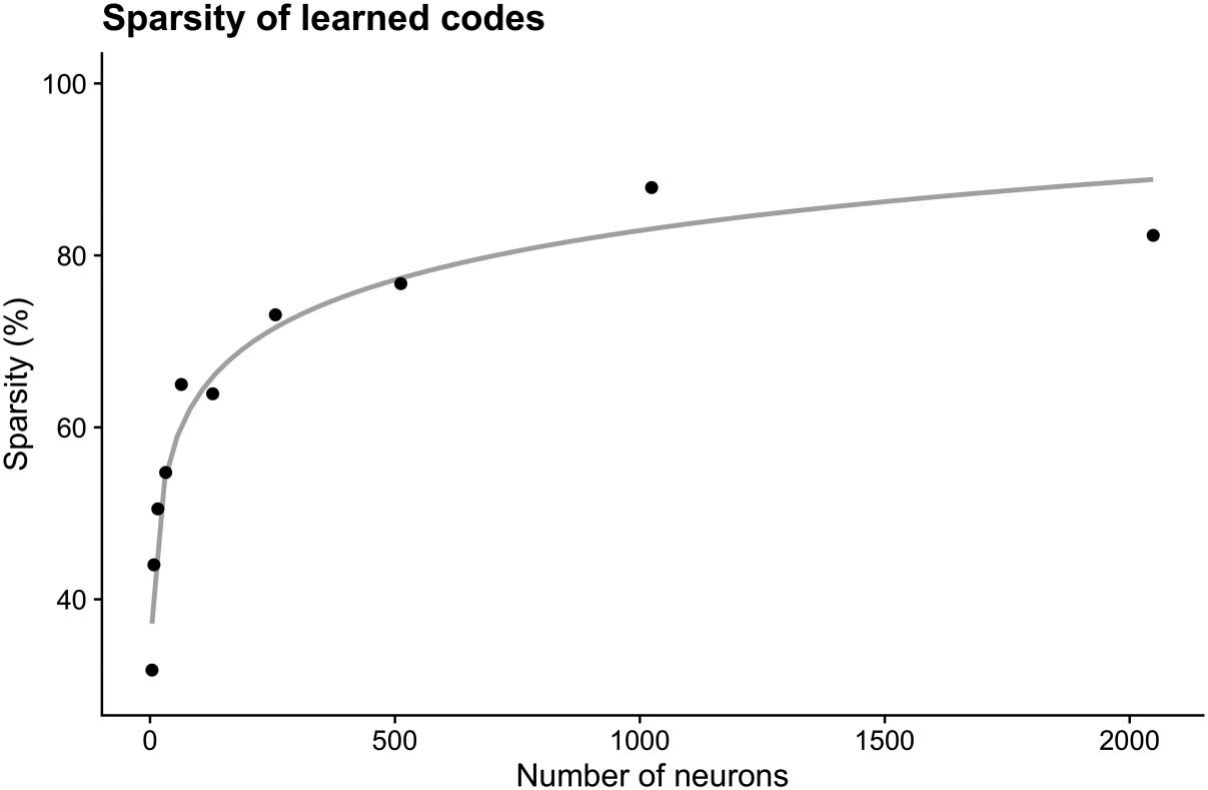
We varied the number of neurons available to the encoder from 2 to 2048 on a logarithmic scale. For each number, we trained 8 codebooks (or policy networks, in terms of reinforcement learning) to encode 12 discrete messages. We thresholded each neural activation at 5% of the maximum firing rate. We then calculated the number of ‘active’ neurons for each message encoding and averaged across codebooks with the same number of neurons. The gray line is a logarithmic curve. We found that the sparsity of learned codebooks is roughly logarithmic with the number of neurons available for encoding.

We began the learning process with randomly initialized codebooks, in which each symbol was mapped to a random vector of firing rates thresholded by a maximum firing rate, which corresponds to a power threshold common in information-theoretic analyses. We found that following RL-based optimization, average transmission time decreased over 100× when compared to randomly initialized codebooks. We also found that a smaller proportion of neurons was used to encode messages as the population of available neurons increased. To characterize this relationship, we thresholded firing rates at 5% of the maximum possible firing rate and computed a percentage of ‘activated’ neurons, averaged over messages and learned codebooks. We found that code sparsity increases roughly linearly with the logarithm of the number of available neurons, as shown in Figure 10. While the investigation reported here is only preliminary, the finding that optimal coding schemes are sparse has an intriguing parallel with neural sparse coding of sensory inputs (Olshausen & Field, 2004).

## DISCUSSION

The model we introduced in this paper provides a generative mechanism for producing a wide range of phenomena found in the study of human response times. Using a series of simple simulations, we demonstrated that characteristic features of human response times, including the Hick-Hyman law, the power law of practice, and the Stroop effect, are mirrored by the time it takes to infer symbols encoded in an array of Poisson processes. The model is not a detailed description of neural dynamics, but rather an intentionally simple algorithm-level model^3^ of inference by an ideal observer. The model describes the minimum time it could possibly take to decode messages encoded in a rate code. Though the human brain is many orders of magnitude more complex than the simple model used in this analysis, the brain’s use of rate codes to encode task-relevant information suggests that human response times might be profitably understood as reflecting the time-course of inference using rate codes. Our investigation showed that a wide range of seemingly disparate phenomena can be parsimoniously understood as necessary features of this simple inference process.

Perhaps the most frequently used model of response time is the drift diffusion model (DDM), which models the accumulation of evidence via a log-likelihood ratio as a Wiener process (Ratcliff et al., 2016). The DDM is widely used to fit response time distributions in Two-alternative Forced Choice (2AFC) tasks, with interpretable parameters that can be easily fit to data. The model is most widely applied to tasks in which the stimulus is stochastic, such as the random dot-motion task (Britten et al., 1992), with the variance of response times interpreted as the result of stimulus noise. In this paper, we interpret noise as intrinsic to the encoding process itself, and accordingly the model predicts variance in response times even with non-noisy stimuli (see also Genest et al., 2016 for supporting neural evidence).

The DDM can be understood as a continuous-time version of Wald’s Sequential Probability Ratio Test, introduced in Wald (1945). Wald assumed that noisy observations were produced by one of two underlying states, and his task was to determine which state was more likely to have generated the observations. Only afterward was Wald’s model adapted to the analysis of human behavior, where it has had astonishing success at modeling 2AFC tasks. Our model is a re-investigation of Wald’s question that uses entropy rather than likelihood ratios to capture decision confidence (it is worth noting that Shannon had not yet published *A Mathematical Theory of Communication* when Wald developed his test). We accordingly consider it a model of ‘information accumulation’ by the decoder rather than ‘evidence accumulation.’

The application of information theoretic concepts to inference in a non-standard way is a key contribution of our model. The model explicitly treats a collection of Poisson processes as a noisy channel over which a message can be transmitted by modulating the processes' rates. The decoder maintains a codebook and updates its uncertainty in real-time as spikes are observed. Entropy is the appropriate quantification of message uncertainty in this context, as it captures subjective uncertainty and is scalable to any number of possible symbols. Entropy computed over the decoder’s posterior belief additionally provides a natural place for leveraging learned task statistics to optimize decoding times, which is critical for reproducing both the Hick-Hyman results and the power law of practice. As applied in engineered systems, information-theoretic models of signal transmission typically maximize efficiency and minimize error by grouping adjacent sequences of symbols and encoding them in batches (Cover & Thomas, 2012). Luce and Laming are right to question how this methodology could possibly provide a useful framework for analyzing information transmission in the brain, reliant as it is on combining multiple messages together for transmission. Our encoding mechanism, on the other hand, transmits one message at a time, while still allowing the decoder to leverage task statistics. We interpret this finding as an indication that Luce and Laming correctly identified an incompatibility in one *implementation* of information theoretic concepts to human behavior, but that the wholesale disregard of information theoretic ideas is not warranted.

### Limitations and Future Work

The results described in this paper were produced by simulations with brute-force sweeps to find parameter values that fit human data. Unfortunately, we do not yet have a closed-form relationship between model parameters and expected decoding times. Such a relationship would facilitate easier model fitting to experimental data and Bayesian model comparisons with competing approaches. We intend to perform such a comparison in subsequent work.

In addition, the model has several simplifying assumptions that could be relaxed. For example, we assume that the decoder knows when the transmission of each symbol begins, with decoding time computed as the difference between the transmission start and the time the entropy threshold is reached. It would be worthwhile to investigate an alternative model in which the decoder continually observes a series of spikes, waiting for a message. We also assume that the decoder maintains a full list of possible messages in the codebook, but we could consider a mechanism for learning the codebook via observations. Finally, while we have provided an analogy between our model and the channel model of transmission, the relationship should be formalized, with the nature of concepts such as transmission rate articulated in the new model.

### Conclusion

The goal of this work is to show that several characteristic patterns of response time can be generated by a simple model of continuous-time inference using information-theoretic framing. We have used information theory to provide a parsimonious explanation for a range of behavioral phenomena that were widely assumed to be unrelated. The spirit of this work is aligned with the recent proposal by Zénon et al. (2019) that cognitive costs in a variety of scenarios reflect the costs of information transmission. In a similar way, we suggest that behavioral response times are a reflection of the bounds on information transmission imposed by encoding in a neural rate code. We echo Hick’s original suggestion that human beings can be considered as a type of channel, but we have modified his proposal to eliminate reliance on complex encoding schemes. Furthermore, we demonstrated that the linear relationship between transmission time and the quantity of information transmitted is sound *in principle* via simulation. We hope this model, and the conceptual shift from evidence accumulation to information accumulation that it requires, stimulates renewed interest in information-theoretic analyses of human behavior.

## Notes

^1^ The baseline firing rate reflects neural “noise” present in recurrent dynamics. It also forces decoding times to take the form of a positively skewed distribution rather than an exponential distribution. Without noise, the first spike in a 1-hot encoding would unambiguously reveal the encoded message.^2^ To instantiate the model computationally, the entropy of the decoder is continually updated at a set time interval *dt* = .1 seconds. The only effect of a smaller *dt* is increased precision of decoding times.^3^ Marr’s second level of analysis (Marr, 1982).

## ACKNOWLEDGMENTS

The authors thank the organizers of the “Information Theoretic Principles in Cognitive Systems” workshop at NeurIPS 2022 for creating a venue for rich discussion about early versions of this work and related topics. We thank the anonymous reviewers for thoughtful and thorough feedback. Finally, we thank John Moore for continued encouragement and editing help on short notice.

## AUTHORS CONTRIBUTIONS

S. Thomas Christie: Conceptualization, Data curation, Investigation, Methodology, Software, Visualization, Writing—original draft, Writing—review & editing. Hayden Johnson: Data curation, Software, Visualization, Writing—original draft, Writing—review & editing. Paul Schrater: Conceptualization, Methodology, Supervision.

## FUNDING INFORMATION

This project did not receive grant funding.

## DATA AVAILABILITY STATEMENT

The code used to run simulations and generate plots can be found at https://github.com/tom-christie/transmit/vignettes.
